# Comprehensive Management of Cholesteatoma in Otitis Media: Diagnostic Challenges, Imaging Advances, and Surgical Outcome

**DOI:** 10.3390/jcm13226791

**Published:** 2024-11-11

**Authors:** Cristina Popescu, Renata Maria Văruț, Monica Puticiu, Vlad Ionut Belghiru, Mihai Banicioiu, Luciana Teodora Rotaru, Mihaela Popescu, Arsenie Cristian Cosmin, Alin Iulian Silviu Popescu

**Affiliations:** 1 ENT Doctor Department of Anatomy, University of Medicine and Pharmacy, Discipline of Anatomy, 200349 Craiova, Romania; cristina.popescu@umfcv.ro (C.P.); arsenie_cristian@yahoo.com (A.C.C.); 2 Research Methodology Department, Faculty of Pharmacy, University of Medicine and Pharmacy of Craiova, 200349 Craiova, Romania; 3 Emergency Medicine and First Aid Department, Faculty of Medicine, University of Medicine and Pharmacy “Vasile Goldiș” Arad, 310025 Arad, Romania; 4 Emergency Medicine and First Aid Department, Faculty of Medicine, University of Medicine and Pharmacy of Craiova, 200349 Craiova, Romania; vlad.belghiru@yahoo.com (V.I.B.); mihai.banicioiu@umfcv.ro (M.B.); luciana.rotaru@umfcv.ro (L.T.R.); 5 Department of Endocrinology, University of Medicine and Pharmacy of Craiova, 200349 Craiova, Romania; mihaela.popescu.e@umfcv.ro; 6 Department of Internal Medicine, University of Medicine and Pharmacy of Craiova, 200349 Craiova, Romania; alin.popescu@umfcv.ro

**Keywords:** cholesteatoma, middle ear, imaging, mastoidectomy, tympanoplasty, bone erosion, radiography, CT, MRI

## Abstract

**Background:** This study presents a comprehensive analysis of cholesteatoma of the middle ear, focusing on its clinical presentation, diagnostic imaging, and treatment outcomes. Cholesteatomas are defined by the keratinized squamous epithelium within the middle ear, leading to significant bone erosion, often affecting the ossicular chain and surrounding structures. **Methods:** The study explores various mechanisms involved in cholesteatoma progression, including enzymatic lysis, inflammatory responses, and neurotrophic disturbances. The study conducted a retrospective clinical and statistical review of 580 patients over a 20-year period (2003–2023), highlighting the role of advanced imaging, including computed tomography (CT) and diffusion-weighted magnetic resonance imaging (DWI), in preoperative planning and postoperative follow-up. **Results:** Findings revealed that early detection and intervention are crucial in preventing severe complications such as intracranial infection and hearing loss. Surgical treatment primarily involved tympanoplasty and mastoidectomy, with a recurrence rate of 1.55% within two years. The study underscores the importance of integrating imaging advancements into clinical decision-making to enhance patient outcomes and suggests further investigation into molecular mechanisms underlying cholesteatoma progression and recurrence. Histopathological and microbiological analysis was performed to identify pathological patterns and microbial agents. **Conclusions:** The study highlights the importance of early diagnosis and intervention to prevent complications such as intracranial infections and permanent hearing loss, while also emphasizing the role of advanced imaging techniques in the management and long-term monitoring of cholesteatoma patients.

## 1. Introduction

Cholesteatomas of the middle ear are characterized by the presence of keratinized stratified squamous epithelium within the cavity, which typically contrasts with the pseudostratified ciliated columnar epithelium found around the Eustachian tube. These lesions are highly erosive, often damaging the ossicular chain, cranial bones, and even the labyrinth, which is considered the most resistant bone in the human body. This destructive potential underscores the severe impact cholesteatomas have on bone tissue. Complete or partial destruction of the ossicular chain is observed in approximately 80% of cholesteatoma cases, compared to only 20% in non-cholesteatomatous chronic otitis media [1].

Cholesteatomas exhibit migratory and lytic properties, which enable them to destroy the ossicular chain and mastoid cell tissue, leading to both intra- and extracranial complications. The precise mechanisms behind cholesteatoma expansion and tissue invasion are not fully understood, but various hypotheses have been proposed. One theory suggests that the erosive phenomena may result from the pressure exerted by the progressive accumulation and retention of epidermal scales. Another potential mechanism involves enzymatic activity, where lytic enzymes secreted by connective tissue cells in the chorion of the matrix contribute to tissue breakdown. Neurotrophic disturbances have also been proposed as contributing factors.

Enzymatic lysis is considered the primary explanation for the destructive effects of cholesteatoma. Walsh theorized that these enzymatic substances originate from the matrix or epithelial debris. Abramson, through histochemical studies, suggested that the erosive factor is hydrolitic, lysosomal, or enzymatic and located in the subepithelial granulation tissue [2]. Additionally, collagenase has been detected in both the cholesteatoma itself and the deep skin of the external auditory canal, further supporting the enzymatic degradation hypothesis.

Regardless of the specific mechanism, it is evident that bone tissue, despite its hardness and resistance, is unable to withstand the invasive nature of cholesteatomas. These lesions can penetrate and erode vast areas of tissue, often leading to significant structural damage and functional impairment in the ear. Recent studies estimate that chronic otitis media, including cases with cholesteatoma, affects approximately 65–330 million people globally, with 60% of these cases resulting in significant hearing impairment. The high incidence in lower-income regions highlights the need for improved diagnostic and treatment strategies. The emergence of antibiotic-resistant pathogens such as *Pseudomonas aeruginosa* and *Staphylococcus aureus* has complicated the treatment of chronic otitis media, contributing to the persistence of infection and the progression to cholesteatoma in untreated cases. Recent data indicate that up to 30% of bacterial isolates from COM cases show resistance to commonly prescribed antibiotics, underscoring the need for effective antimicrobial stewardship. The challenge of antibiotic resistance requires a multifaceted approach, combining precise surgical intervention with targeted antimicrobial therapy to manage chronic otitis media and prevent the development or recurrence of cholesteatoma [3].

The evaluation of cholesteatomas involves several diagnostic imaging modalities aimed at assessing the extent of bone erosion and tissue involvement [4]. Conventional radiography, especially in Schüller’s view, has been widely used to evaluate mastoid pathology. However, while conventional X-rays provide useful preliminary insights, they are limited by their inability to display structures in different planes. This summation of structures into a single image complicates the interpretation and may obscure critical details of the cholesteatoma.

Schüller’s view is particularly valuable in examining the surgical aspects of the middle ear and offers a comprehensive view of the mastoid air cell system. This view is often the first step in the radiological assessment of middle ear pathology, especially in cases of suspected inflammatory processes. Comparative temporotympanic radiographs are essential to obtain an overview of the petromastoid region with additional views chosen based on clinical signs to further investigate the pathology.

In addition to conventional radiography, computed tomography (CT) has become a key imaging tool for evaluating cholesteatomas. CT scans provide high-resolution images that allow for the detailed visualization of bone erosion, including the involvement of the ossicular chain, mastoid cells, and other surrounding structures. This imaging modality is particularly useful for preoperative planning, as it helps delineate the extent of the cholesteatoma and guides surgical intervention.

Magnetic resonance imaging (MRI) can also play a role in the evaluation of cholesteatomas, particularly in differentiating between residual or recurrent disease and postoperative changes. Diffusion-weighted (DWI) MRI is highly sensitive to the presence of keratinous debris, which is characteristic of cholesteatomas. DWI can detect small cholesteatomas and is especially useful for postoperative follow-up to identify residual or recurrent lesions that may not be visible on CT scans. Endoscopic ear surgery has emerged as a promising minimally invasive technique that provides better visualization of cholesteatoma, especially in anatomically challenging areas, reducing the need for extensive mastoidectomy and preserving hearing structures [5].

Surgical removal is the primary treatment for cholesteatomas, given their destructive nature and potential for serious complications if left untreated. The goal of surgery is to completely eradicate the cholesteatoma while preserving as much of the normal ear structure and function as possible. Depending on the extent of the disease, different surgical approaches may be employed, including tympanoplasty, mastoidectomy, or a combination of both [6].

Postoperative imaging, particularly with DWI, is critical for monitoring for recurrence, as cholesteatomas have a tendency to recur even after complete surgical excision. Long-term follow-up is essential to ensure that any residual or recurrent disease is identified and treated promptly to prevent further complications.

The prognosis for patients with cholesteatomas largely depends on the extent of the disease at the time of diagnosis and the success of surgical intervention. Early detection and complete removal of the cholesteatoma offer the best chances for preserving hearing and preventing more severe complications, such as intracranial infections or facial nerve paralysis. However, in cases of advanced disease with significant bone erosion, some degree of permanent hearing loss or other complications may be unavoidable. The study uniquely integrates the use of DWI to detect residual and recurrent cholesteatoma, demonstrating a higher diagnostic accuracy compared to traditional imaging techniques. Our findings show that DWI is particularly effective in postoperative follow-up, allowing for early detection of disease recurrence that might not be visible on standard CT scans. The integration of advanced imaging techniques, particularly DWI, into the diagnostic and preoperative planning process can enhance the early detection of cholesteatoma and improve surgical precision. This approach reduces the need for repeat surgeries and supports better long-term outcomes for patients. This approach exemplifies how integrating advanced imaging can lead to better surgical planning and reduced postoperative complications. Current literature often lacks a clear consensus on the best imaging practices for monitoring cholesteatoma recurrence. Our study addresses this gap by providing data that supports the use of DWI as a superior tool for early detection of residual disease, which could lead to the development of standardized imaging protocols [7].

This study aims to provide a comprehensive analysis of cholesteatoma in the context of chronic otitis media, focusing on the diagnostic challenges, advancements in imaging modalities, and the effectiveness of surgical interventions. By integrating a retrospective analysis of clinical cases, this work seeks to identify key factors influencing diagnostic accuracy and treatment outcomes, thus contributing to more effective management strategies for patients with this condition. The study hypothesizes that advanced imaging techniques, such as CT and MRI, improve diagnostic accuracy and surgical planning, leading to better treatment outcomes and reduced recurrence rates.

## 2. Materials and Methods

### 2.1. Study Design

This retrospective clinical-statistical study was conducted over a 20-year period (2003–2023) at the ENT Clinic of the County Emergency Clinical Hospital Craiova. The study focused on patients diagnosed with acute and chronic otic pathologies.

### 2.2. Study Population

A total of 580 patients aged 7–70 years were included, selected from hospitalized patients in the clinic.

#### 2.2.1. Inclusion Criteria

- Patients aged 7–70 years diagnosed with acute or chronic middle ear conditions
- Individuals who underwent diagnostic imaging (X-ray, CT, and MRI) and surgical treatment (tympanoplasty and mastoidectomy).
- Patients with complete clinical records and consent for data use in research.
- Patients who have available follow-up data for at least two years post-surgery to assess outcomes.

#### 2.2.2. Exclusion Criteria

- Patients with incomplete clinical or imaging data.
- Individuals without confirmed middle ear pathology through clinical or radiological assessment.
- Cases where ear symptoms were not primarily related to otic pathologies.
- Patients with significant comorbid conditions (e.g., immunosuppression, systemic diseases) that may impact surgical outcomes or recovery.

### 2.3. Diagnostic Protocols

Patients followed an otic symptomatology assessment protocol in order to plan and evaluate the outcomes of medical and particularly surgical treatment. Therefore, patients were questioned in detail about each symptom they presented.

The determining signs for the diagnosis were recorded in all patients of the group, being represented by: date of onset and duration of auricular suppuration, appearance and quantity of otic secretion, size of the external auditory canal and appearance of the tympanum (congestive infiltrated tympanum, presence of pulsatile pus visible through the tympanal perforation), condition of the retroauricular tissues, auditory function, alterations in general condition (fever, fatigue, insomnia, pallor). The external auditory canal and tympanic membrane were examined otoscopically for changes in color and luster, changes in normal anatomical relief, pathological changes in the surface, thickness and continuity of the tympanum, and changes in tympanic mobility. The collection of auricular secretions for the identification of pathogens was carried out by wiping with a cotton-tipped stylet, and the storage and transportation of the collected secretions were carried out under strictly sterile conditions, according to known procedures.

### 2.4. Imaging Techniques

Imaging was integral to the clinical decision-making process at multiple stages of patient management. Initially, CT scans were employed to assess the extent of bony involvement and identify structural changes such as ossicular erosion. Findings from CT helped determine the need for more extensive surgical approaches, including mastoidectomy in combination with tympanoplasty. Additionally, DWI was used to detect soft tissue lesions and residual disease, which informed the extent of tissue removal during surgery. Postoperatively, DWI allowed for early detection of any recurrence, ensuring timely intervention.

X-rays of the petromastoid region were taken in all patients under study, using the Schüller temporo-tympanic incidence compared bilaterally, this incidence being the first examination in middle ear exploration, particularly in inflammatory processes. X-rays were performed on a classic ELTEX 400 radiodiagnostic X-ray machine, with 18/24 X-ray films. The patient was placed in the ventral decubitus position, with the chin tucked far to the chest, the head placed in perfect profile, with the sagittal plane parallel to the box, on which he was supported with the external auditory orifice placed 2 cm above the middle of the box; and the ear flap had to be turned forward to avoid overlapping the mastoid cells. The central ray angled cranio-caudally by 25–300 entered through a point located at 7 cm cranially and slightly posterior to the external auditory opening of the ear on the tube side and exited through the external auditory opening on the opposite side.

CT scans were performed on a total of 168 patients, using both native and post-contrast imaging with intravenous iodinated contrast, while carefully considering any absolute contraindications. The technique involved examination in the supine position with acquisition in the axial plane. Both soft tissue and bony structures were taken into account in image interpretation. Computed tomography scanning was performed with HITACHI CT PRESTO, software version 3.02, with fine sections, 1 to 2 mm pitch, photon flux of 300–700 mA, and voltage of 110–130 kV.

MRI was performed on a total of 128 patients, using specific protocols for the petromastoid region, with acquisition in three planes, T1 and T2-weighted, native and post-gadolinium-weighted. Scanning was performed using GE Signa 1T and AIRIS Mate HITACHI, 0.2 T, software version V4.5U. The examination protocol included T1- and T2-weighted spin-echo sequences, with FOV 200, matrix 200 × 200, range 5.0, and thickness 3.

Mucosal fragments from the middle ear or mastoid cavities were harvested during surgery and sent for examination to the pathology laboratory, where classical histologic staining (hematoxylin-eosin and trichrome) and immunohistochemistry staining were performed. In all patients, histopathologic examination of the polyp, cholesteatoma, or mucosa fragments collected intraoperatively was performed.

### 2.5. Audiometric Assessment Protocol for Preoperative and Postoperative Hearing Evaluation

Audiometric assessments were conducted using Grason-Stadler GSI-61 audiometers calibrated annually. Hearing thresholds for air and bone conduction were measured at standard frequencies (500 Hz, 1 kHz, 2 kHz, and 4 kHz) in a soundproof booth. The pure-tone average (PTA) was calculated by averaging these thresholds, and the air-bone gap (ABG) was determined by the difference between air and bone conduction. Masking was applied when necessary to prevent cross-hearing. Postoperative audiometric assessments were performed at 6 months to evaluate hearing outcomes. Improvement was defined as a ≥10 dB reduction in PTA or a ≥15 dB reduction in ABG.

### 2.6. Statistical Analysis

To evaluate the relationship between preoperative imaging findings and postoperative outcomes, a statistical analysis was conducted using data from 580 patients who underwent tympanoplasty and mastoidectomy. Imaging results from preoperative CT scans and postoperative DWI were compared with key outcomes, including cholesteatoma recurrence, hearing improvement (measured by audiometry), and the occurrence of postoperative complications.

The analysis began with descriptive statistics, which provided a summary of continuous variables such as patient age, duration of symptoms, and the extent of bone erosion, as well as categorical variables such as the presence of residual disease and complications. Frequency distributions were used for categorical data, while means and standard deviations were calculated for continuous variables.

Next, univariate analyses were performed to explore the relationship between specific imaging findings (e.g., the extent of ossicular damage or labyrinthine fistulae) and postoperative outcomes. Chi-square tests were used to analyze categorical outcomes, while t-tests assessed differences in continuous variables between groups of patients with or without postoperative complications or hearing improvement.

To account for potential confounders, a multivariate logistic regression model was applied. The postoperative outcomes (recurrence, hearing improvement, and complications) served as the dependent variables, and key imaging findings (such as bone erosion, ossicular damage, and residual disease detected on DWI) were the independent variables. Age, sex, and symptom duration were adjusted for in the model to control for confounding factors. A *p*-value of less than 0.05 was considered statistically significant, and odds ratios (OR) with 95% confidence intervals (CI) were calculated for each predictor variable.

## 3. Results

### 3.1. Analyzing the Results of Radio-Imaging Investigations

#### 3.1.1. Conventional Radiology

The radiographic features captured are multiple and complex depending on the degree of mastoid cell damage and the stage of the disease.

Out of the total number of patients examined in the early stage, 68 patients were included, in whom radiography showed weak and uniform mastoid cell voiding. A number of 40 patients showed demineralization of the intercellular septa with preservation of the macrostructure of the intercellular walls, which corresponds to a later stage of the disease.

In 116 patients, conventional radiography visualized extensive osteolysis with the appearance of osteochondensation and demineralization in adjacent bone structures.

In 360 patients, the radiological examination revealed images of geodes accompanied by osteosclerotic changes and a complete lack of air cell development in all mastoid cells, and in 136 patients, the lateral venous sinus was visualized. The radiologic examinations of all patients showed a low percentage of 18.49% who were in the early stage of the disease, from the radiologic point of view; in stage II, the percentage of patients was 29.86%, and the largest proportion of the studied group, 61.64%, was in stage III, which revealed once again that the patients’ referral to the doctor was late.

The complexity and multitude of radiographic features were determined by the evolution of the inflammatory and infectious processes that occurred in the petromastoid region, depending on the stage of the disease.

The inflammatory process gradually decreased the normal transparency of the mastoid region. The changes in transparency were given by the appearance of serous or purulent exudate that replaced the air in the cell system; later, the exudate could be replaced by granulation tissue, the changes in transparency being even more evident.

The early stage in this pathology showed a poor and uniform voiding of the mastoid cells.

**Figure 1:**
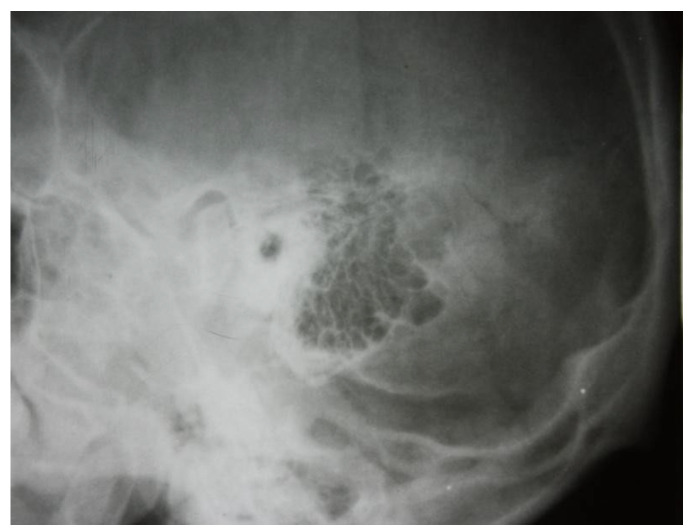
A radiograph of the left petromastoid region in the temporo-tympanic (Schüller) incidence showed reduced transparency of most mastoid cells.

**Figure 2:**
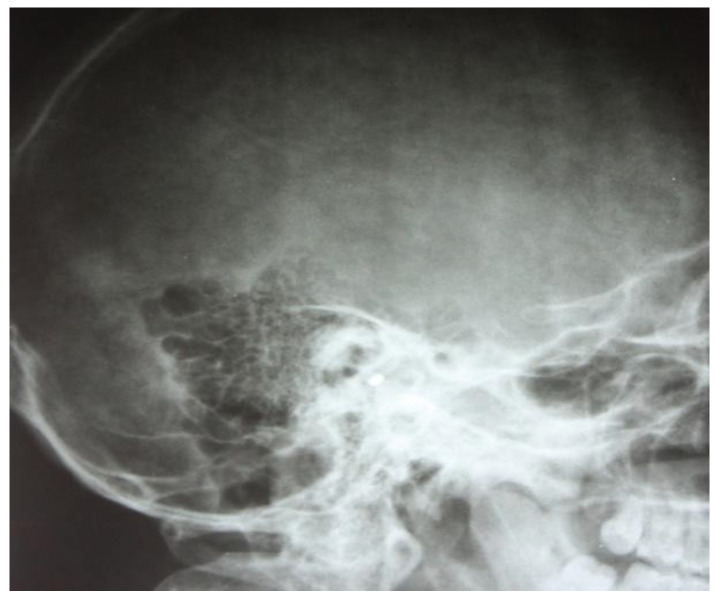
Reduced pneumatization of mastoid cells, with preservation of the bony septa between the cells.

**Figure 3:**
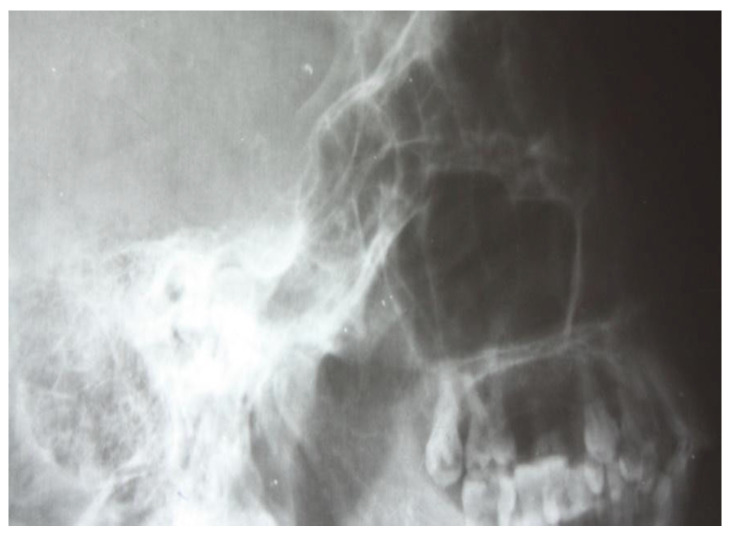
Pneumatic cells with demineralized intercellular septa, with a faded, mottled, punctiform appearance, in places losing their individuality (accentuation of local vasomotor phenomena). There is no evidence of missing or defective bone structure.

The two images (Figure 1 and Figure 2) are suggestive for showing the reduction in transparency of the majority of mastoid cells, with the maintenance of intercellular septa, without showing any lack or defect of bone structure (Figure 3).

**Figure 4:**
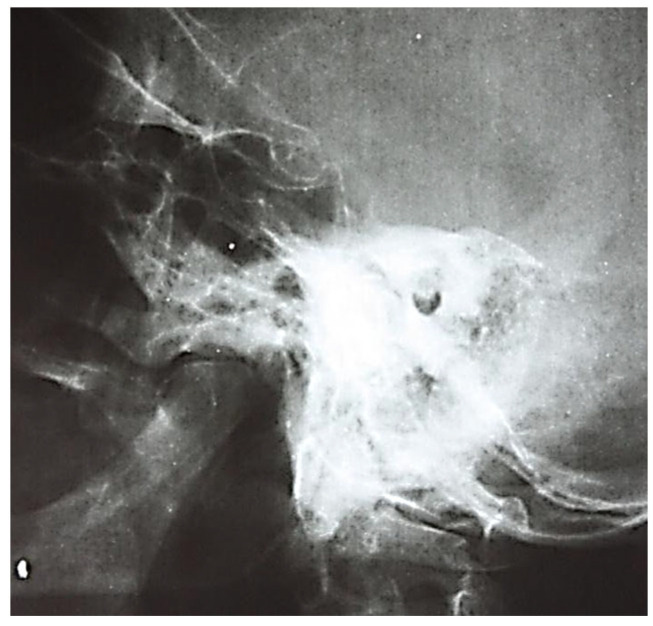
Elements to reduce pneumatization and resorption of intercellular septa.

At a later stage there was demineralization of the intercellular septa, the septa appearing thinner with increased transparency, the radiological appearance being erased or stained, and the pathophysiological support being local vasomotor phenomena. However, at this stage, the macrostructure of the intercellular walls has not been disrupted; clinically, this phase corresponds to the transformation of the serofibrinous exudate into purulent; the following image (Figure 4) emphasizes these aspects.

The demineralization of the intercellular septa may be the only change in bone structure evidenced, together with the reduction in transparency; these aspects set in before any other radiologically visible change in bone structure.

In the advanced stages of inflammation, the transparency of the middle ear cavity was greatly reduced, resulting in an intense and homogeneous opacity with the appearance of granulation tissue; the chain of ossicles gradually lost its individuality until complete disappearance.

**Figure 5:**
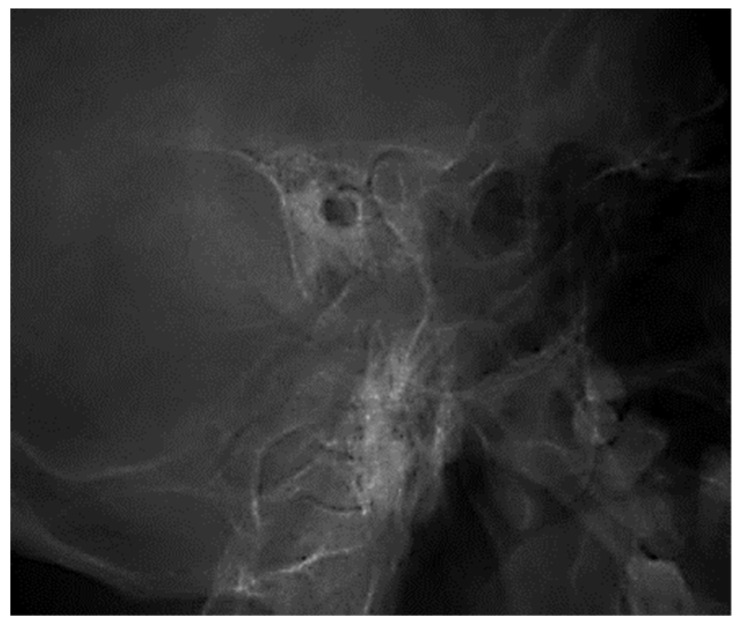
Small areas of osteolysis at the petrous portion, more evident anteriorly of the posterior margin of the temporal rock.

**Figure 6:**
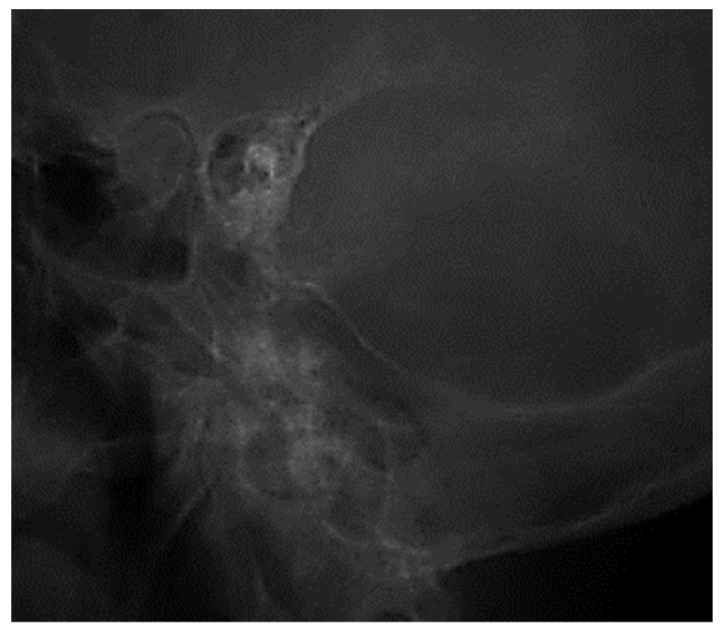
Small geode projected at the petrous region.

In this regard, the following images (Figure 5) emphasize the tendency to loss of individuality and transparency of the mastoid cells, with the appearance of osteochondensation elements and the faded, blurred appearance of the residual pneumatized cells. The evolution of the pathological process was accompanied by osteolytic changes, with the radiograph showing disrupted septa and extensive demineralization in adjacent structures. Osteolysis is the result of bone resorption by osteoclasis, radiologically evidenced by a more or less intense lack of bone substance, translated by areas of increased transparency, usually with an irregular outline (Figure 6).

We also observed accentuation of the intensity of the usual limits of some anatomic elements, such as the lateral venous sinus or tegmen tympani. However, extensive osteonecroses were found in infections with increased virulence, occurring in the entire mastoid block or in groups of pneumatic cells with a tendency to confluence.

**Figure 7:**
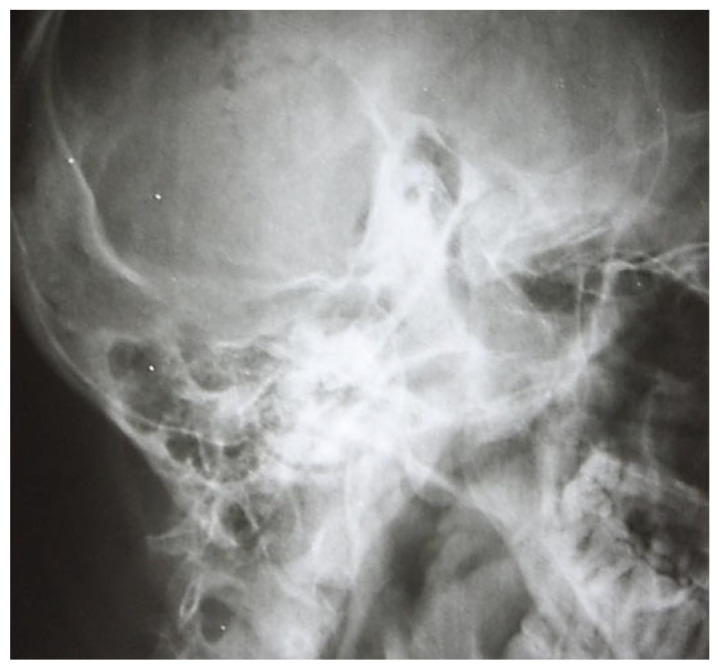
Postoperative control radiograph showing an area of osteolysis with clear, well-demarcated borders.

**Figure 8:**
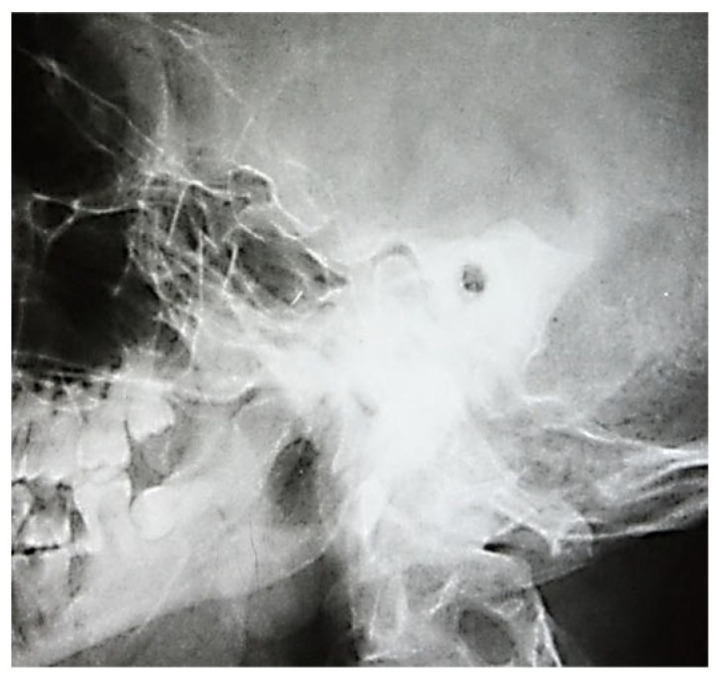
Osteocondensation of the petromastoid region with complete disappearance of pneumatization and evidence of the lateral venous sinus posterior to the posterior margin of the temporal rock.

**Figure 9:**
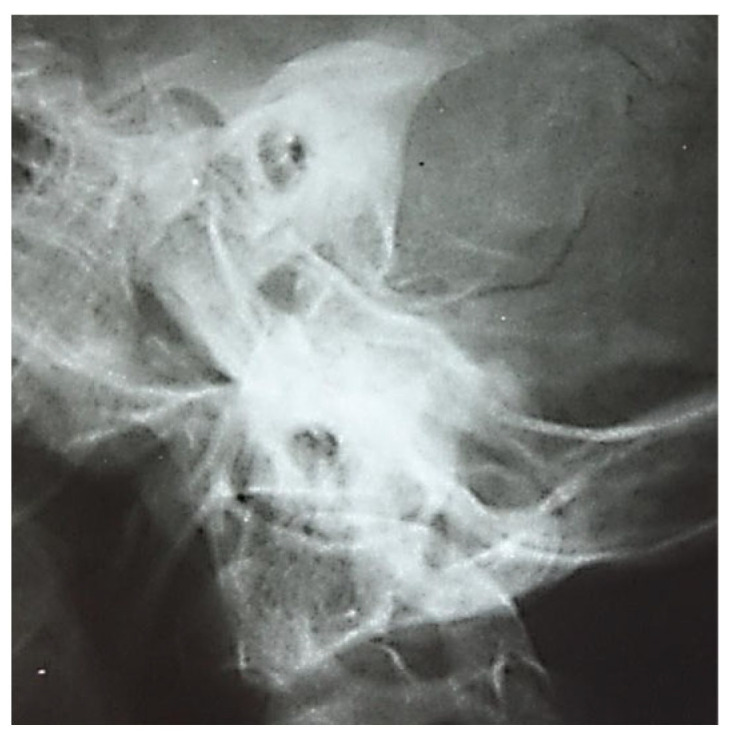
Total loss of mastoid aeration accompanied by marked bone condensation changes (sclerosis).

**Figure 10:**
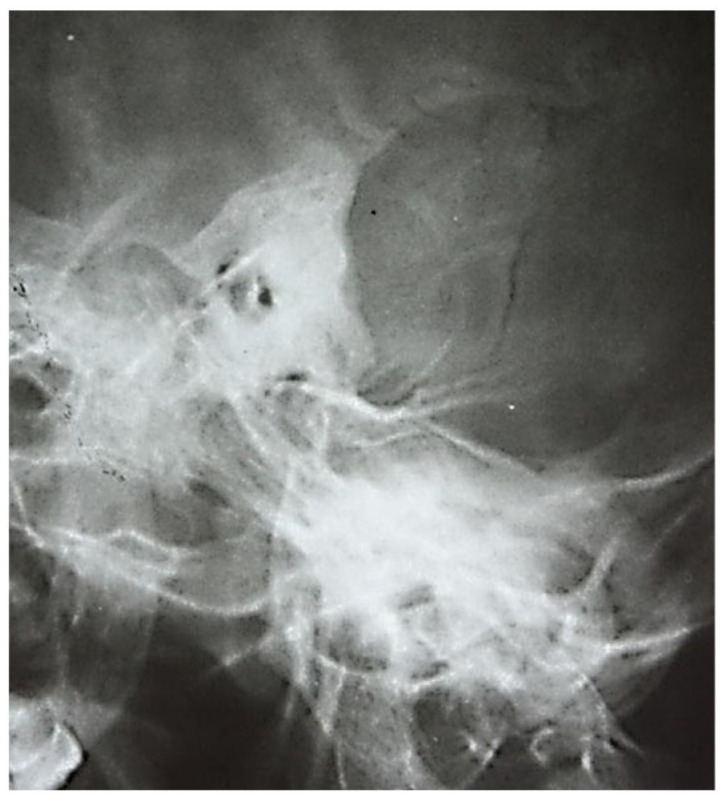
Lack of mastoid air spaces in the pre- and retrosinusal cells, with visualization of the lateral venous sinus (band of increased transparency, 1–1.2 cm wide, with smooth borders, oblique from top to bottom, parallel to the posterior border of the rock).

At the postoperative control examination, the radiographic image showed an area of osteolysis, oval, projected in the anterior portion of the temporal rock, with smooth, well-demarcated borders (Figure 7). Chronic otic chronic inflammatory processes have as a repercussion osteosclerosis that dominates the radiological picture and may extend from the antral and periantral region to the entire mastoid; osteosclerotic changes may be accentuated, their intensity being influenced by the age of the pathological process; radiographically, the anatomical elements of the region can be visualized with great difficulty (Figure 8, Figure 9 and Figure 10).

#### 3.1.2. Computed Tomography and Magnetic Resonance Imaging

Out of the studied group, computed tomography was performed in 168 patients (28.76%) and magnetic resonance imaging in 128 patients (21.91%). Both imaging methods were used in 96 patients (16.43%).

An image suggestive of cholesteatoma was seen in 68 patients, accompanied by adjacent osteolysis and fluid (Table 1 and Table 2).

**Figure 11:**
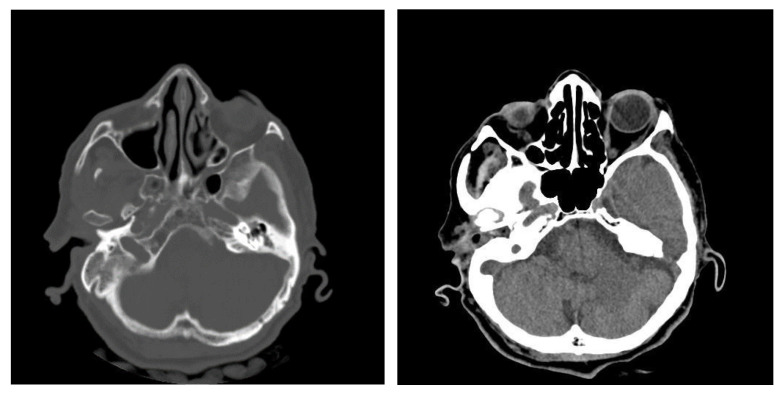
Patient with diagnosis of right suppurative chronic polypous polypous otomastoiditis. CT examination in axial sections shows absence of mastoid cell pneumatization and presence of a tissue mass in the right external auditory canal.

In this retrospective study, patients with chronic otic pathologies commonly presented with symptoms such as otodynia, fetid mucopurulent otorrhea, and varying degrees of hearing loss. These symptoms typically had an onset that extended over several months, with some patients experiencing progressive worsening shortly before seeking treatment. Clinical examinations frequently identified mucopurulent secretions in the external auditory canal, and otoscopy often revealed polypoid formations within the epitympanum. CT played a critical role in the assessment, with scans showing reduced mastoid aeration in the affected cells, indicating chronic inflammatory changes. CT images also commonly revealed masses with tissue densities, along with fluid and parafluid collections within the external auditory canal, providing detailed visualization of the extent and nature of the disease. These imaging findings were essential for diagnosing the condition and planning appropriate surgical interventions. (Figure 11). Following the correlations made between the clinical and imaging aspects, the diagnosis of chronic right suppurative polypous otomastoiditis was established, in addition to the interpretation of the tonal audiogram.

**Figure 12:**
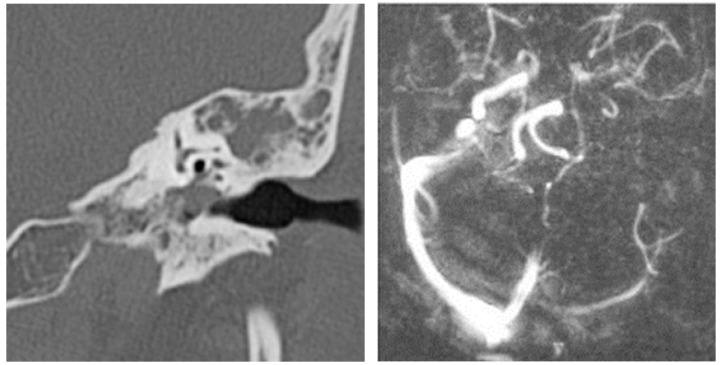
Patient with diagnosis of chronic suppurative otomastoiditis, lateral venous sinus thrombosis. CT scan, axial section, bony window showing absence of pneumatization of left-sided mastoid cells, fluid retention in the area, and MR venous 2DTOF MRI venous 2DTOF sequence with absence of signal at the left-sided lateral venous sinus.

**Figure 13:**
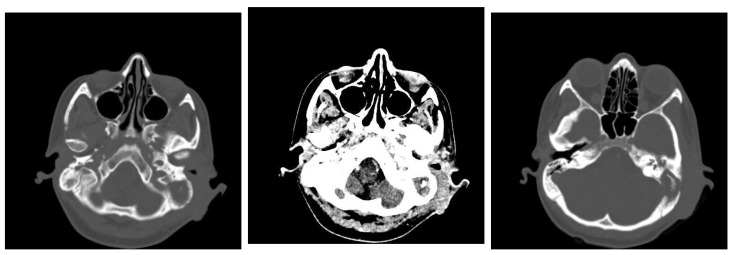
Patient with the diagnosis of chronic suppurative left exteriorized otomastoiditis. CT examination, in postcontrast CT axial sections, parenchymal window and bone window, with bone sequestration and fistulization in the subcutaneous soft parts.

In pediatric patients, otomastoiditis was often characterized by acute episodes that could rapidly progress to complications. Children commonly presented with systemic infectious symptoms, such as fever and general malaise, along with localized signs of ear infection. Clinical examinations frequently revealed retroauricular infiltration of the soft tissues, while otoscopic evaluations identified reddish polypoid formations prolapsing through the epitympanic perforation, accompanied by grayish-yellow, viscous purulent secretions. CT images of these cases typically showed poorly developed mastoid air spaces, the presence of tissue formations within the external auditory canal, and diffuse infiltration of the retroauricular soft tissues, extending into the adjacent subcutaneous fat (Figure 12). Another frequently encountered complication in chronic otomastoiditis was lateral venous sinus thrombosis. CT examination showed a lack of pneumatization of the left mastoid with fluid accumulation in this area (Figure 13).

**Figure 14:**
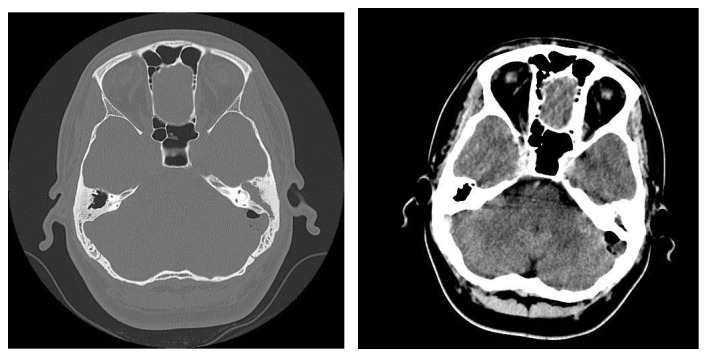
Patient with diagnosis of chronic suppurative otomastoiditis, brain abscess. CT examination in axial sections, bone window, and parenchymal window, post-contrast, showed lack of pneumatization of left mastoid cells; in the brain substance adjacent to the posterior aspect of the left temporal rock, gas bubbles and diffuse and moderate contrast uptake.

In cases of advanced chronic otomastoiditis, local complications were frequently observed, particularly in patients presenting with severe or septic conditions. Emergency CT examinations, both native and post-contrast (with intravenous iodine), often revealed critical features such as bony sequestration surrounded by detritus, moderate contrast uptake, and signs of osteolysis with fistulization of the external trabecula. Additionally, infiltration of the retroauricular soft tissues, including the adjacent skin and tissue around the external auditory canal, was commonly detected. Diagnosis was established based on a combination of clinical findings and patient history, including conditions such as viral hepatitis, visceral leishmaniasis, and hematologic disorders that contributed to an immunosuppressed state. Surgical intervention, such as radical petromastoidectomy, was performed to evacuate the suppuration and manage the complications. Postoperative outcomes were generally favorable, with primary healing achieved, and follow-up visits confirmed sustained recovery, even in patients with complex medical backgrounds. (Figure 14).

**Figure 15:**
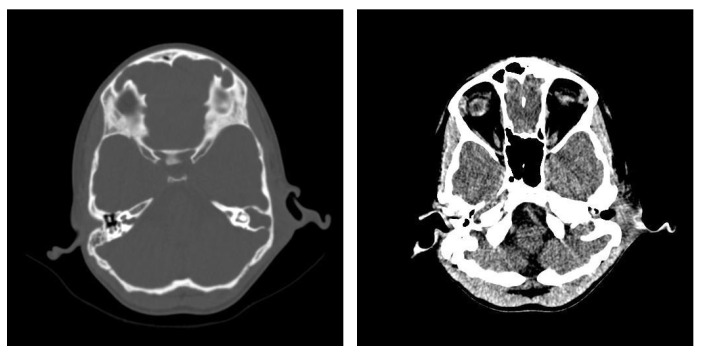
Patient with the diagnosis of acute suppurative polypous exteriorized retroauricular acute polypoid otomastoiditis. CT examination in axial, bony window, and parenchymal sections revealed underdeveloped mastoid air spaces on the left side, tissue formation in the external auditory canal, and diffuse infiltration of the retroauricular soft tissues.

One of the most serious complications associated with chronic otomastoiditis is brain involvement, which can manifest as intracranial infections. Patients typically presented with acute neurological symptoms such as fever, headache, nausea, vomiting, and dizziness, often developing rapidly. Clinical history often revealed repeated episodes of chronic suppurative otitis media, with exacerbations triggered by concurrent infections. In cases where neurological symptoms were present, computed tomography (CT) scans were crucial for diagnosis. CT imaging frequently showed a lack of pneumatization in the mastoid cells, along with parafluid densities. In more severe cases, imaging revealed findings suggestive of an infectious process in the brain, such as gas bubbles and diffuse, moderate contrast uptake in the brain parenchyma adjacent to the temporal bone. These features indicated possible brain abscesses and adjacent cerebritis, requiring immediate medical intervention (Figure 15). Bilateral mastoid involvement was found in a total of ten patients, seven of whom were in the age group up to 20 years.

In patients with chronic otomastoiditis, symptoms often developed slowly, with a range of associated auditory and nasal issues. Common findings on otoscopic examination included bloody-purulent secretions in the external auditory canal, which, when cleared, revealed mesotympanic perforations and hyperemic, granular, or polypoid-transformed mucosa covered with purulent secretions that obscured anatomical landmarks. Bilateral examination sometimes showed features such as a thin, translucent tympanic membrane molded onto the stapes, indicating structural changes linked to hearing loss.

**Figure 16:**
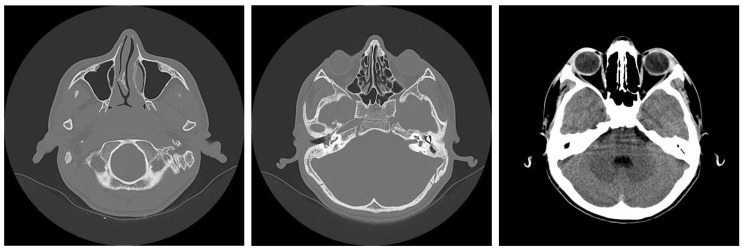
Patient with the diagnosis of right chronic suppurative polypous chronic polypous otomastoiditis, left chronic otomastoiditis, deviated nasal septum, and chronic hypertrophic rhinitis. CT examination, axial sections, bone, and parenchymal window—hypertrophy of bilateral middle nasal turbinates, accentuated on the left side; reduced pneumatization of mastoid cells on the left side; lack of air cell development in the mastoid cells; presence of effudion with increased densities in the right mastoid.

**Figure 17:**
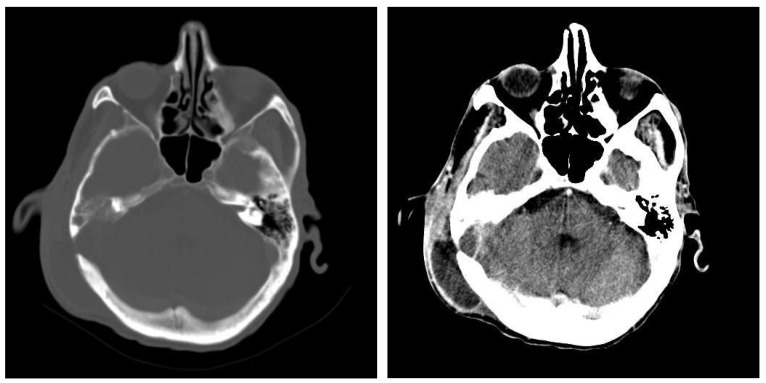
CT axial sections, bone, and parenchymal window showing right temporooccipital osteolysis and at the posterior aspect of the temporal rock on the right side, right temporooccipital epicranial collection and diffuse infiltration of the integument and retroauricular fat, right cerebellar subdural collection with subdural empyema appearance.

**Figure 18:**
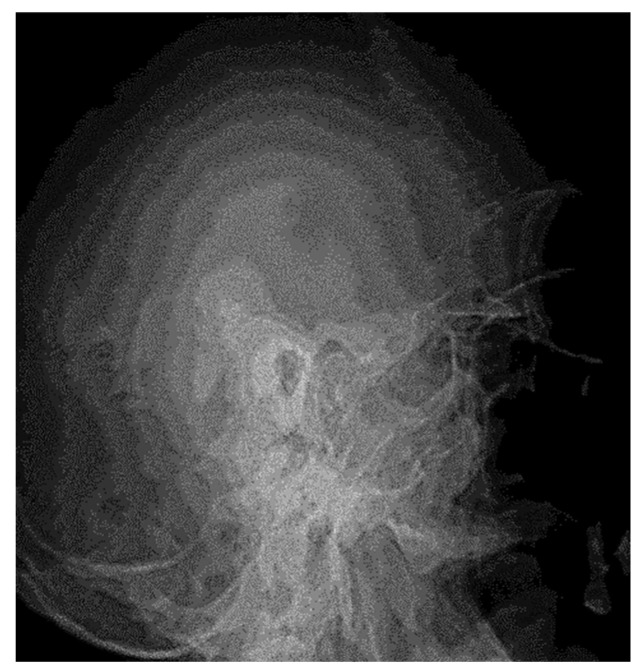
Left petromastoid region, Schüller’s incidence shows reduced left-sided mastoid pneumatization.

Additional diagnostic evaluations, such as nasal endoscopy, frequently identified complications such as septal deviation, septal perforations, and hypertrophy of the middle turbinate, which could obstruct the nasal passage. Radiographic and CT findings supported these observations, revealing osteocondensation of the petromastoid regions, opacification of the pneumatic system, and reduction in mastoid cell pneumatization. On the affected side, imaging often demonstrated high-density fluid in the mastoid, the presence of tissue masses in the middle ear, and osteocondensation of the ossicular chain, consistent with conductive hearing loss. Bilateral audiograms commonly indicate conductive hearing loss, aligning with these structural abnormalities. CT imaging further confirmed conditions such as turbinate hypertrophy and reduced mastoid cell pneumatization, correlating with patients’ reports of hearing impairment. Despite these findings, there was no evidence of pathological contrast enhancement in the brain parenchyma, suggesting no intracranial extension in these cases (Figure 16). The presence of retroauricular swelling, which represents an exteriorization and therefore a complication of the disease, required a computed tomography examination, with the results of image interpretation demonstrating both retroauricular exteriorization but also bone destruction and especially the existence of an intracerebral complication—posterior fossa subdural empyema (Figure 17). Radiographic examination performed in Schüller’s incidence revealed reduced mastoid pneumatization on the left side (Figure 18).

**Figure 19:**
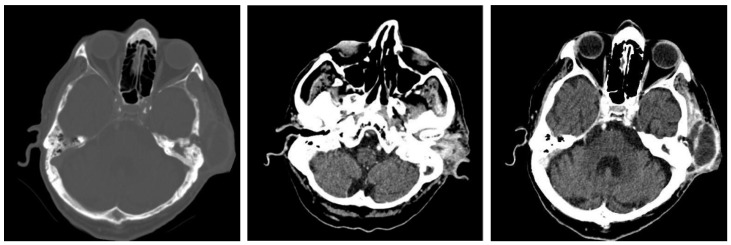
Axial CT sections, bone and parenchymal window—extensive area of osteolysis in the left temporal bone and external wall of the mastoid, absence of pneumatization of left-sided mastoid cells and reduced pneumatization of right-sided mastoid cells, epididymal collection, diffuse infiltrative appearance of the preauricular soft tissues.

A computed tomography examination revealed an extensive area of osteolysis in the bone window at the level of the left temporal bone and external wall of the mastoid, with the presence of bone sequestrations, complete loss of mastoid air spaces on the left side, and reduced pneumatization of the right-sided mastoid cells. Postcontrast examination revealed diffuse infiltrative appearance of the preauricular soft tissues with extension to the temporomandibular joint and an image of an epicranial collection with a temporal starting point and extension to the parietal level; diffuse infiltration of the left temporal muscle was also evidenced (Figure 19).

**Figure 20:**
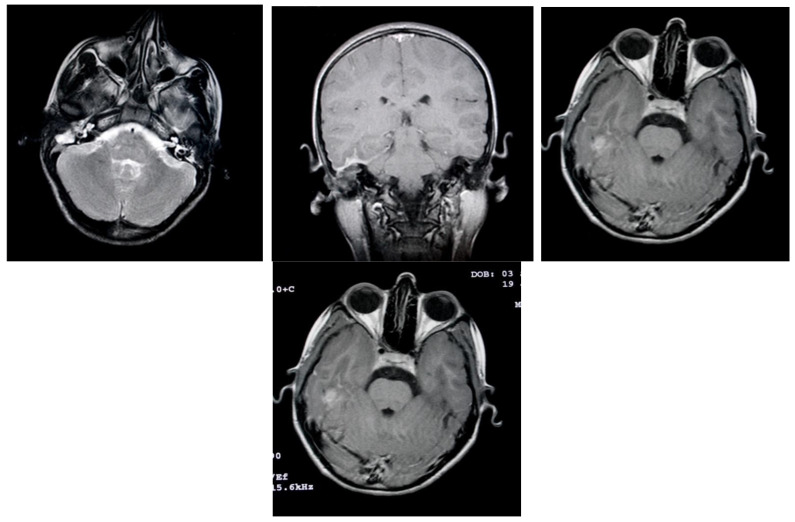
Patient with the diagnosis of acute cholesteatomatousotomastoiditis, right temporal cerebritis, and neighboring meningeal reaction in MRI examination with axial and coronal sections native and postgadolinium, an area in frank hyperseminal at the right mastoid, with fluid appearance; postgadolinium, diffusely demarcated area, at the level of the brain parenchyma, right temporal lobe, and meningeal pathologic contrast uptake, right temporal.

Among the cases studied, 64 patients experienced an unfavorable postoperative course, which included complications requiring further management. Patients diagnosed with acute cholesteatomatous otomastoiditis were occasionally found to have intracerebral inflammatory or infectious processes. Initial MRI examinations in these cases revealed signs suggestive of intracranial involvement, such as localized inflammation or abscesses. These imaging findings highlighted the potential for serious complications and emphasized the importance of comprehensive diagnostic evaluation and prompt treatment to prevent the spread of infection beyond the ear structures. (Figure 20).

**Figure 21:**
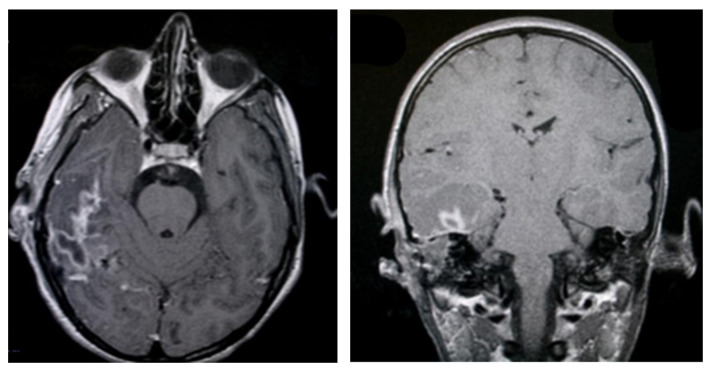
Axial and coronal postgadolinium axial and coronal sections show right temporal cerebral abscesses and pathologic uptake of neighboring meningeal contrast.

**Figure 22:**
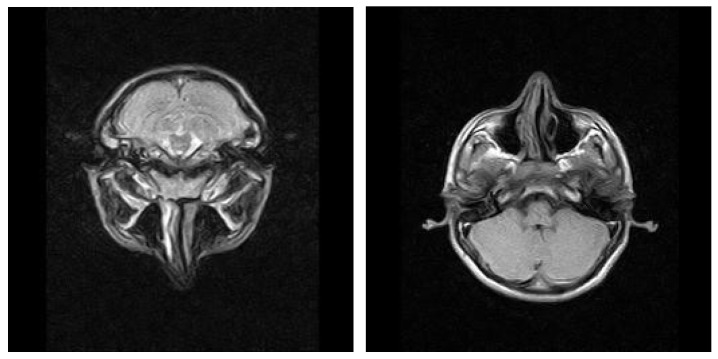
Axial T2-weighted and T1-weighted MRI T2-weighted and T1-weighted MRI axial sections show hypersignal T2-weighted area, isosignal with the T1-weighted brain substance, located in the right middle ear; also, heterogeneous signal area is observed in the right mastoid, indicating the presence of superinfected fluid.

MRI examinations often revealed a hypersignal area in the mastoid region, with fluid characteristics suggestive of mastoiditis. Post-gadolinium imaging commonly showed diffuse, abnormal contrast uptake in the cerebral parenchyma, particularly in the temporal lobe, and along the temporal meningeal region. These findings were indicative of complications such as cerebritis and meningeal reactions. Despite emergency surgical intervention and the initiation of specific drug therapy, some patients experienced an unfavorable clinical course, marked by persistent fever and focal neurological symptoms. Follow-up MRI examinations in these cases revealed the development of brain abscesses in the temporal lobe and continued abnormal meningeal contrast uptake, confirming the progression of the intracranial infection, and necessitating further medical management. (Figure 21). In cases of complicated otomastoiditis, native MRI examinations often revealed distinctive imaging characteristics. T2-weighted sequences typically showed hypersignal areas that were isosignal with T1-weighted brain substances, indicating abnormalities within the middle ear. Additionally, heterogeneous signal areas were frequently observed in the mastoid region, suggesting the presence of superinfected fluid. (Figure 22).

**Figure 23:**
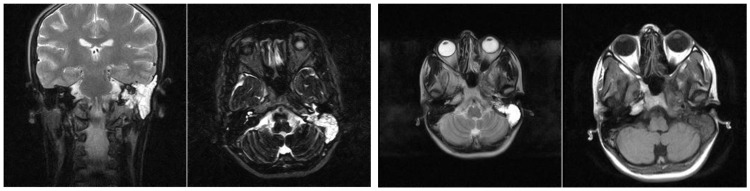
Patient with the diagnosis of chronic suppurative left retroauricular exteriorized chronic suppurative otomastoiditis, MRI examination with axial and coronal sections, with enlarged hyperseminal T2-weighted area in the middle ear and mastoid on the left side, hyposeminal T1-weighted.

Imaging studies frequently demonstrated the extension of infectious processes from the middle ear and mastoid to surrounding regions, including the retroauricular area. MRI examinations were particularly valuable in detecting such complications, showing infiltration of adjacent tissues and tegument. Patients with a history of recurrent acute otitis media, often treated with prolonged courses of broad-spectrum antibiotics, were at risk of developing tympanic perforations with further extension of infection. The presence of general systemic symptoms and clinical suspicion of retroauricular involvement typically warranted advanced imaging to confirm the extent of the disease and inform appropriate treatment (Figure 23).

**Figure 24:**
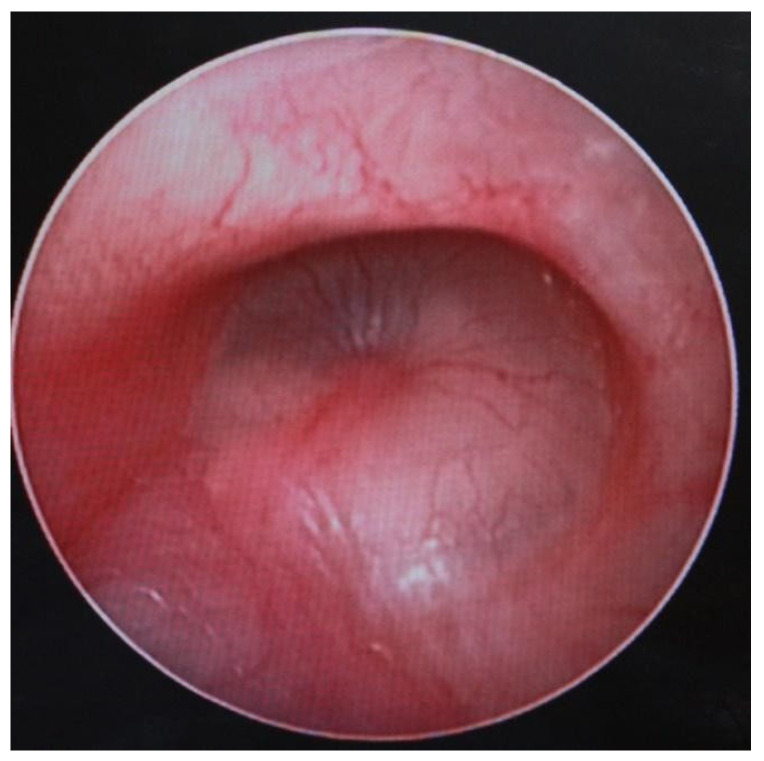
Endoscopic examination of the right tympanic membrane with slight accentuation of the tympanic vascularization.

MRI findings in patients with chronic otomastoiditis often showed enlarged T2-weighted hypersignal areas within the middle ear and mastoid, indicating fluid accumulation or inflammation. Corresponding T1-weighted images typically revealed hypointense areas, consistent with the fluid accumulation or infection, without evidence of involvement in the cerebral or cerebellar parenchyma. Despite these imaging findings, endoscopic examination frequently confirmed that the tympanic membrane remained intact, as observed in several cases. This combination of imaging and endoscopic assessments provided a comprehensive view of the extent of the disease, aiding in diagnosis and management (Figure 24).

The results show that a significant majority of the patients (82.76%) presented with hearing loss, with 70.83% of cases being conductive in nature, typical of conditions such as cholesteatoma, which affects sound transmission in the middle ear. The remaining 29.17% had sensorineural hearing loss, indicating some level of inner ear involvement. The mean preoperative PTA was 55 dB (±10 dB), which falls into the moderate hearing loss range, suggesting considerable hearing impairment before surgery. The mean ABG of 25 dB further confirms the predominance of conductive hearing loss in this cohort, showing a substantial gap between air and bone conduction thresholds (Table 3).

All 580 patients who underwent surgery received a combination of tympanoplasty and mastoidectomy. The success rate for tympanoplasty in this study was 81.89%, with most patients achieving stable outcomes without significant complications. In our cohort, 11.03% of patients experienced severe complications following mastoidectomy. No cases of recurrence were detected during the first postoperative year, indicating the initial effectiveness of the surgical intervention and thorough disease removal. However, 9 cases (1.55%) of recurrence were identified within the first two years postoperatively (OR = 3.35, 95% CI: 0.20–55.24, *p* > 0.08). 70% of patients experienced significant improvements in hearing thresholds post-surgery, measured through audiometry. Univariate analysis showed that patients with extensive ossicular damage on preoperative CT were significantly more likely to experience postoperative hearing loss (*p* < 0.05). Additionally, the presence of residual disease on postoperative MRI was strongly associated with cholesteatoma recurrence (*p* < 0.001). Patients with labyrinthine fistulae demonstrated a higher likelihood of postoperative complications, such as hearing deterioration or persistent infection, compared to those without (*p* < 0.02). In the multivariate logistic regression analysis, several imaging findings were identified as strong predictors of postoperative outcomes. Patients who had ossicular chain damage visible on preoperative CT scans were found to be 2.8 times more likely to experience hearing loss after surgery (OR = 2.8, 95% CI: 1.4–5.6, *p* < 0.003). Furthermore, patients with labyrinthine fistulae identified on imaging had a 3.5-fold increased risk of developing postoperative complications, including persistent infections or further hearing deterioration (OR = 3.5, 95% CI: 1.5–6.9, *p* < 0.02) (Table 4).

The postoperative results indicate that 70% of patients experienced improved hearing following surgery, with a ≥10 dB improvement in PTA in 406 individuals. Additionally, 17.24% had unchanged hearing, while 12.76% experienced worsened hearing postoperatively. The mean postoperative PTA was 35 dB (±8 dB), reflecting a significant improvement in hearing thresholds compared to preoperative levels. The postoperative ABG was reduced, with a mean ABG of 15 dB, showing improved conductive hearing. Notably, 51.72% of patients achieved an ABG ≤ 10 dB, indicating near-complete restoration of conductive hearing, while 20.69% still had an ABG > 20 dB. Furthermore, 31.03% of patients experienced complete closure of the ABG, meaning their conductive hearing loss was fully resolved after surgery (Table 5).

#### 3.1.3. Analysis of Histopathologic and Immunohistochemical Results

The histopathologic analysis demonstrated that cholesteatoma was present in 276 patients, indicating a significant incidence of this destructive middle ear condition. Additionally, 180 patients exhibited polyps, a marker of chronic inflammation commonly associated with middle ear pathology. Furthermore, in 128 patients, the tympanic membrane mucosa showed the presence of apocrine-like cells and chronic inflammatory infiltrate, reflecting ongoing inflammation and pathological changes in the middle ear. These findings underscore the dual nature of the disease process, involving both destructive and inflammatory components in patients with chronic otitis media (Table 6).

The histopathological study revealed, in 47.58% of the cases of the studied batch, a much modified mucosa, consisting of a keratinized squamous epithelium, close in structure to the epidermis. The thickness of the epithelium varied from one case to another and even from one area to another of the same case. The macroscopic appearance of hypertrophy of the tympanic mucosa corresponds microscopically to mucipital hyperplasia and metaplasia. The hyperplasia of the mucosa translates into an increase in the number of cells in both the covering epithelium and the underlying chorion. At the level of the chorion, the hyperplasia caused the appearance of lymphoplasmacytic infiltrate and angiogenesis vessels. Mucomatous metaplasia occurs due to the transformation of the unistratified epithelium into a pseudostratified cylindrical respiratory-type epithelium.

**Figure 25:**
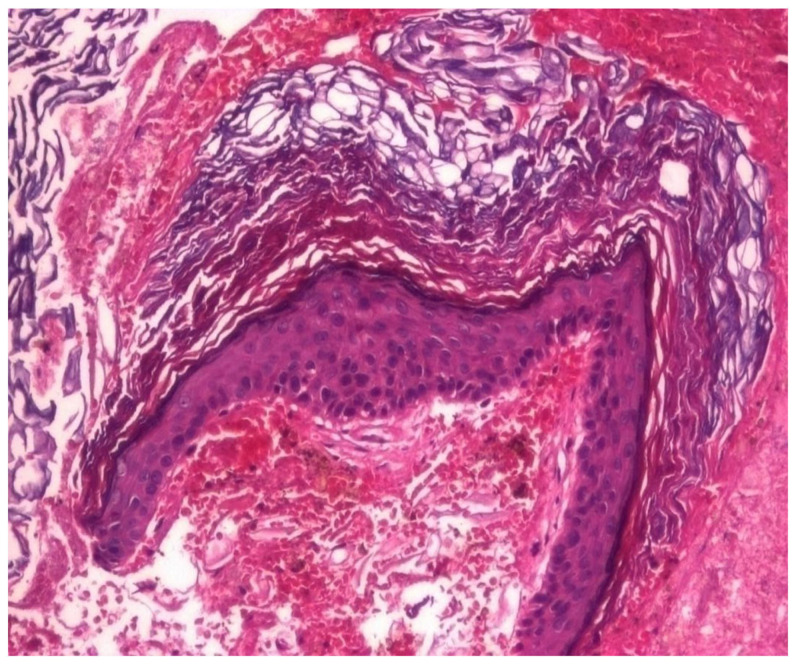
Cholesteatom, ob. ×10, col. HE.

**Figure 26:**
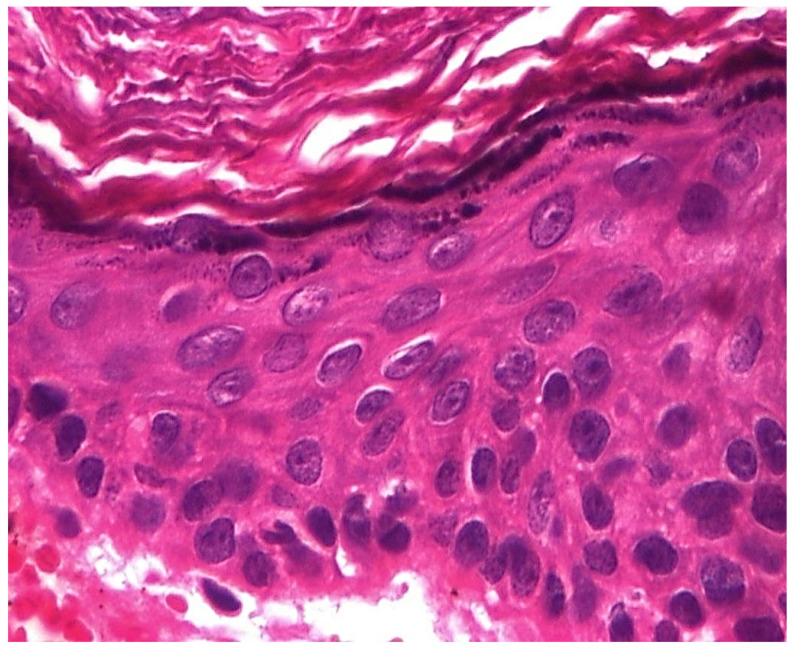
Cholesteatom, ob. ×40, col. HE.

In the structure of the epithelium, four layers of cells were evident: with round, hypochromatic, nucleolated nuclei. Large intercellular spaces, crossed by numerous desmosomes, were evidenced between the cells; a layer consisting of 2–5 rows of rhomboidal cells, with basophilic keratohyalin granules (Figure 25 and Figure 26).

**Figure 27:**
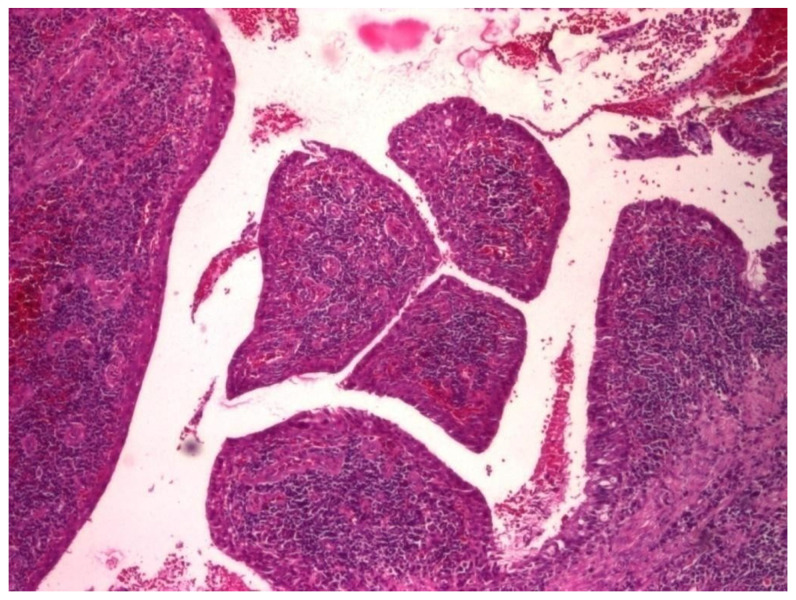
Polip, ob. ×4, col. HE.

**Figure 28:**
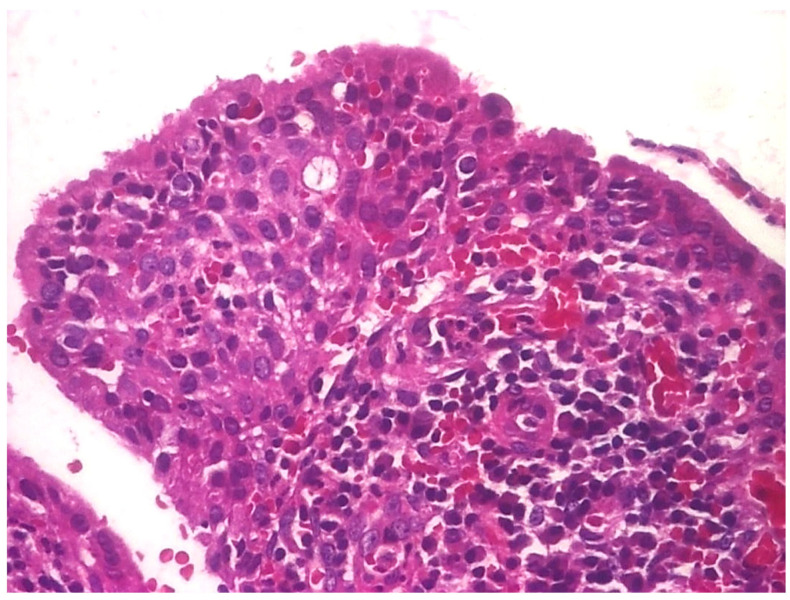
Polip, ob. ×20, col. HE.

In 31.03% of cases, the histopathology study revealed microscopic structures of the polypous type (Figure 27 and Figure 28).

Thus, on histological sections, several conical, protruding formations were identified, delimited at the periphery by a pseudostratified cylindrical epithelium with a dense cellular stroma, richly vascularized, with numerous angiogenesis vessels.

In some cases, small areas of necrosis were evident in the stroma of these polypoid formations.

**Figure 29:**
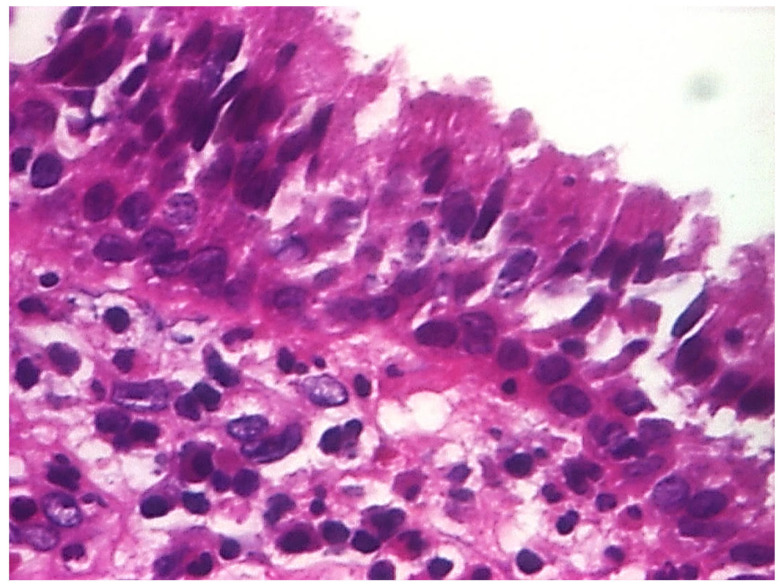
Tympanic membrane mucosa, epithelium with apocrine-like cells and chronic inflammatory infiltrate, ob. ×40, col. HE.

**Figure 30:**
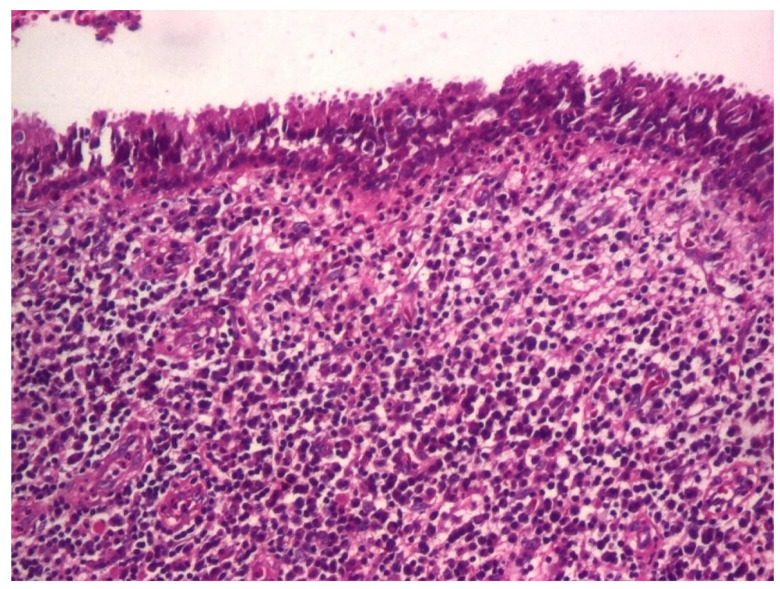
Tympanic membrane mucosa, epithelium with apocrine-like cells and chronic inflammatory infiltrate, ob. ×10, col. HE.

In 22.06% of cases, histopathologic study revealed the presence of a mucosa of the tympanic chamber with altered epithelium, numerous apocrine cells, and a chorion richly infiltrated with lymphoplasmacytic cells (Figure 29 and Figure 30). Sometimes, extensive areas of epithelial necrosis or, on the contrary, areas of hyperplasia and hypertrophy of the covering epithelium were revealed.

Immunohistochemical examination of intraoperatively collected fragments was performed in 248 patients.

**Figure 31:**
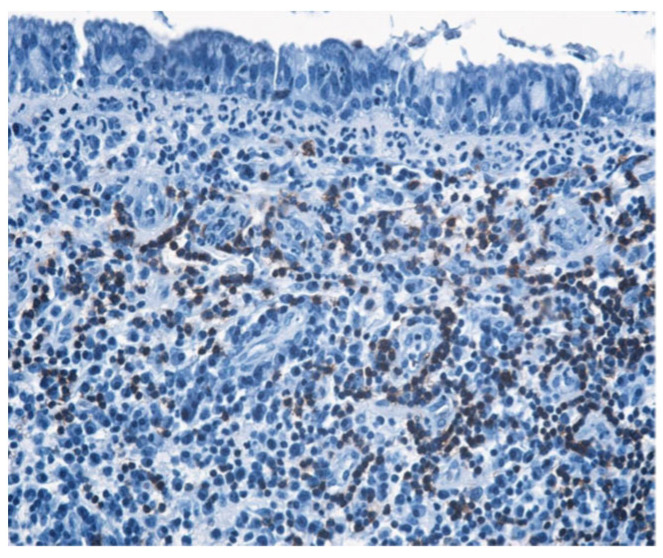
B lymphocytes in small amounts diffusely distributed, highlighted by IHC technique using CD20 atc, ob. ×100.

**Figure 32:**
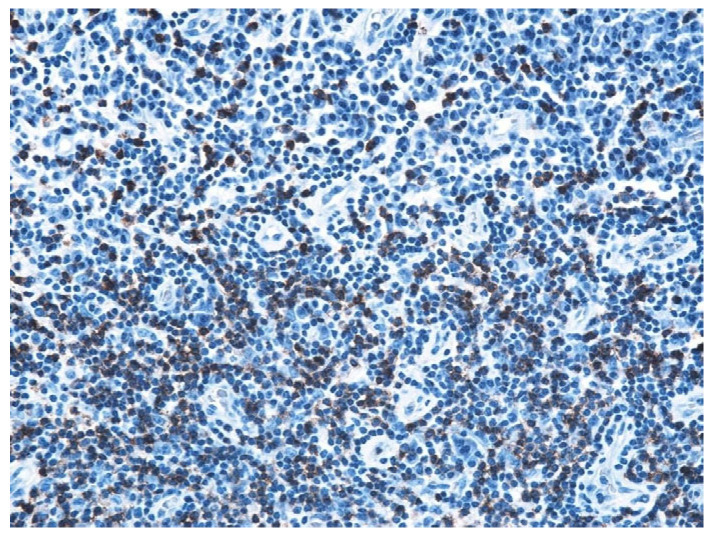
Much more numerous T lymphocytes arranged around blood vessels, ob. ×100.

**Figure 33:**
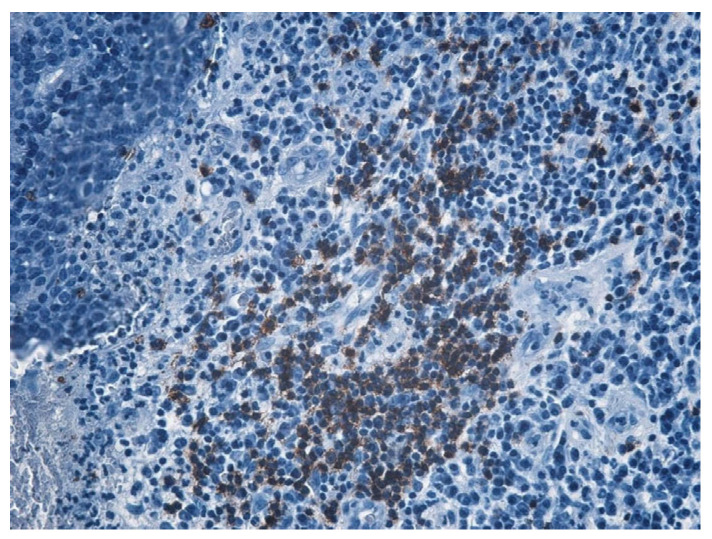
T lymphocytes unevenly distributed, more abundant around blood vessels, granuloma-like appearance; T lymphocytes visualized by IHC technique using atc CD3, ob. ×100.

**Figure 34:**
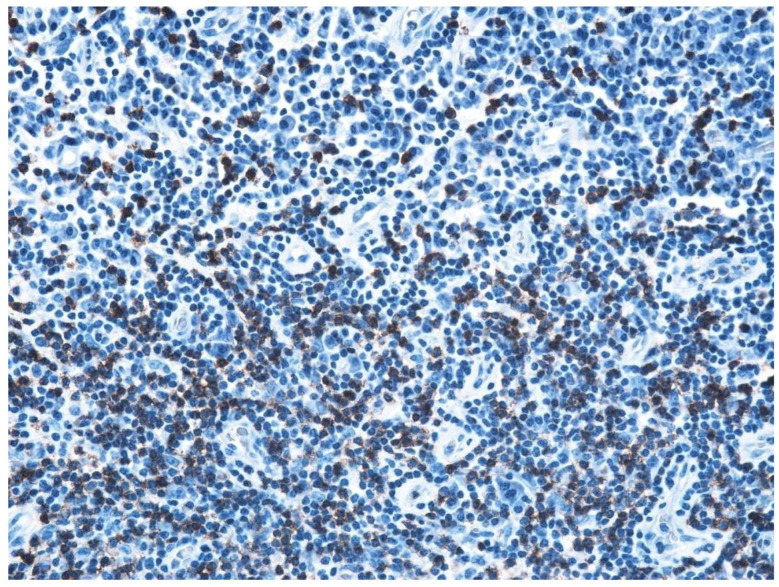
Much more numerous T lymphocytes arranged around blood vessels, ob. ×100.

**Figure 35:**
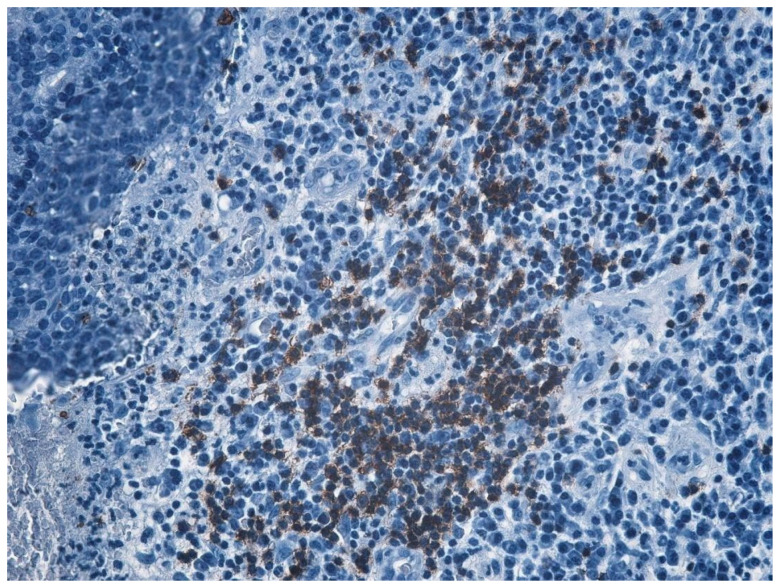
T lymphocytes unevenly distributed, more abundant around blood vessels, granuloma-like appearance; T lymphocytes visualized by IHC technique using atc CD3, ob. ×100.

The immunohistochemical study revealed the cell types present in the inflammatory stromal infiltrate: T lymphocytes, B lymphocytes, and macrophages, with a non-homogeneous distribution (Table 7, Figure 31, Figure 32, Figure 33, Figure 34 and Figure 35).

## 4. Discussion

Chronic suppurative otitis media continues to pose a significant challenge, especially in developing countries, where diagnosis and effective management remain difficult. Although the incidence of complications has significantly decreased due to better use of antibiotics, these infections remain a critical issue in the context of inadequate healthcare systems and a lack of public awareness. Chronic suppurative otitis media complications can be classified into intracranial and extracranial types, with the most common being otitic meningitis, lateral sinus thrombosis, and brain abscesses, which require prompt surgical intervention to prevent serious sequelae or death. Early diagnosis and timely surgical management are essential to improving the prognosis of patients with these severe complications [8,9,10]. Despite advancements in treatment, the accurate diagnosis of otitis media remains a significant challenge. Studies indicate that misdiagnoses are common, leading to inappropriate treatment and contributing to the problem of antibiotic resistance. Improving diagnostic skills and ensuring better access to functional equipment are critical measures to address this ongoing public health concern [11].

The histopathologic features observed in the study correspond with other data in the medical literature. Normally, the mucosa of the tympanic chamber and mastoid cells consists of a simple columnar epithelium, pseudostratified in places, with tall, ciliated cells and mucus-secreting cells (predominantly arranged around the orifice of the eustachian tube) and a reduced chorion, consisting of lax connective tissue with numerous round mononuclear cells, belonging to the immune system [12,13]. In chronic otitis media, prolonged inflammation thickens the mucosa, reducing tympanic cavity spaces, potentially leading to granuloma formation. Mucosal metaplasia occurs, transforming simple endothelium into respiratory-type epithelium with mucus-secreting goblet cells and ciliated cells, impacting the middle ear’s clearance and drainage functions due to excessive secretions and microbial toxins. Although reversible with early treatment, chronic cases can cause irreversible tissue damage, including inflammatory granulomas and bone lesions, leading to chronic suppurative otitis media. This condition features inflammatory polyps, necrosis of ossicles, and histological changes, resulting in progressive hearing loss due to impaired middle ear function [14].

Studies in the field [15,16] on the long-term evolution of otomastoiditis treated medically in outpatient clinics have found a discrepancy between clinical symptoms and radiologic appearance, the persistence of pathologic radiologic features persisting long after the clinical symptoms have subsided, thus justifying repeated radiologic examinations.

Walsh T.E., in his work on the effects of cholesteatoma on bony structures, mentions the time lag between clinical symptoms and radiologic images [17].

Also of particular importance is the correct execution of radiography and comparative analysis with the appearance of the normal mastoid on the opposite side, especially in the early stages, when the change in bone structure is due only to demineralization of the intercellular septa.

The radiologic examination allows an evolving follow-up of the acute infectious inflammatory process, capturing all its phases. Some recent articles [18,19] consider that osteosclerosis may also be the result of delayed therapy or unjustified discontinuation of therapy and that the degree of osteosclerosis and the age of the inflammation may also be correlated, with the most intense condensation of the region being seen in old, long-standing otomastoiditis. In the radiologic interpretation of a mastoid X-ray of a previously operated patient, performed in Schüller’s view, a differential diagnosis between the presence of cholesteatoma and postoperative bone loss is essential. This distinction is achievable as cholesteatoma appears with well-defined borders and condensed contours, while osteonecrosis presents with blurred, irregular edges. Post-surgical changes exhibit well-defined radiologic boundaries [20]. The advantages of conventional radiology include simplicity, low cost, and widespread availability of standard radiologic equipment in most hospitals. However, its limitations arise from the summation of structures in a single plane, complicating image interpretation. Radiologic assessment should begin with comparative temporotympanic radiographs for an overview of the petromastoid region, followed by additional views based on clinical signs. The Schüller view is particularly useful for examining the surgical aspect of the middle ear, offering a comprehensive view of the mastoid air cell system. As this view highlights crucial anatomical features of the petromastoid region, it should be the primary imaging technique in evaluating middle ear pathologies, especially inflammatory processes. We consider that the microscopic changes observed by us in otomastoiditis were generated both by the inflammatory process and through the mediators released by the cells and by the presence of microbial flora.

In the present study, the most numerous anatomoclinologic forms of otomastoiditis were cholesteatomatous. This form of otomastoiditis can occur in both children and adults, but there appear to be differences in clinical manifestations, course, and recurrence according to age [21]. Cholesteatomatomas can be considered benign tumors characterized by an abnormal growth of cells of the lining epithelium lining the middle ear and mastoid cavities, associated with complex and dynamic changes in the chorion cells and extracellular matrix [22,23].

According to some authors [24], the occurrence of chronic cholesteatomatous chronic otomastoiditis is thought to be due to a lack of control of cell proliferation in the covering epithelium, which leads to the development of an epidermoid cyst (cholesteatoma) characterized by the appearance of a keratinized stratified squamous epithelium within the pneumatized areas of the temporal bone. The fact that some cholesteatomas are congenital has led some authors to speculate that the lack of control of cell proliferation may be due to abnormalities in the genes that control proliferation. Cytokines released by inflammatory cells following recurrent otitis media also appear to be involved in the development of cholesteatoma [25,26].

In addition to the local inflammatory process, the cholesteatoma itself appears to play a major role in the development of complications since, through enzymatic bone resorption, it allows the infection to extend outside the ear, leading to endotemporal, extra- or intracranial suppurative complications. The mechanisms of bone destruction by cholesteatoma are not yet fully elucidated. So far, pressure necrosis, the action of perimatrial inflammatory granuloma, chronic osteomyelitis, and the action of osteoclasts and osteocytes stimulated by various local factors produced by the cells of the inflammatory process are identified as possible mechanisms. Thus, histochemical studies have shown that in the inflammatory focus and at the level of the cholesteatoma, local activation of collagenases, phosphatases, proteases, and local pH changes plays an important role in the mechanisms of bone destruction. Collagenases are classified as metalloproteinases. They have been evidenced in the perimatrix of cholesteatases and are thought to be the main factor responsible for the osteolysis process [27,28].

Through immunohistochemical study, we sought to highlight the cell types present in the inflammatory stromal infiltrate. Of the immune system cells present, the most numerous were T lymphocytes, then B lymphocytes, and the least numerous were macrophages. The distribution of cells in the inflamed chorion had an inhomogeneous distribution, indicating a varied antigen distribution. In addition, an immunohistochemical study showed that in chronic otomastoiditis, cell-mediated immunity appears to be dominant.

Abramson mentioned that in the perimatrix there is a collagenase with a very high collagenolytic potency, higher than in normal epidermis. This, together with other local factors, would cause bone lysis and spread of infection from the middle ear and mastoid to neighboring tissues, including the endocranium. He found that upon contact between epidermis and connective tissue, there is an acceleration of collagenolytic activity three times greater than in locally separated tissues. Collagenase alone cannot accomplish bone destruction. R. Yuasa’s chemical analysis of the cholesteatomatous content revealed the presence of five volatile fatty acids that may have lytic action on hydroxyapatite crystals.

After demineralization and degeneration of extracellular organic substances, collagen fiber breakdown and bone resorption follow as a result of osteoclast activation.

It can be concluded that the synergistic action of fatty acids and collagenase could have an osteolytic action in perimatrix inflammatory granuloma.

Other microscopy studies performed at the perimatrix have demonstrated intense activity in this tissue, with an increase in local cellularity and in the density of angiogenesis vessels. This angiogenesis favors keratinocyte proliferation, increased enzyme activity at this level, and osteoclast activation, thus explaining bone resorption and centrifugal proliferation of the cholesteatoma. In the situation of the superinfected cholesteatoma, the structure is more difficult to trace, the limiting membrane is perforated, and the layered structure is visible only in small portions; the central part is represented by a gray, amorphous, septate, fetid, septate mass. A section through the infected cholesteatum shows bands of connective tissue rich in coarse granulation, produced by the disintegration of inflammatory cellular elements intricately interwoven with epithelial debris.

The role of microbiota and biofilm in the pathogenesis of cholesteatoma has been highlighted in recent studies. Cholesteatomas often exhibit altered microbiota with a higher abundance of *Staphylococcus* species, particularly *Staphylococcus aureus* and *Pseudomonas aeruginosa*, which are known for their biofilm-forming capabilities. The presence of biofilm contributes to persistent infections and frequent recurrences by providing bacteria with protection against the host immune response and standard antibiotic treatments. In this context, microbiota and biofilm may play a significant role in the progression and recurrence of cholesteatoma, suggesting the need for targeted therapeutic approaches against biofilm-associated resistance [29,30]. Although our study did not include an extensive microbiological analysis, future research should incorporate microbiota profiling and biofilm analysis to better understand the pathophysiology of cholesteatoma and improve treatment strategies.

This study has several limitations that should be acknowledged. A retrospective analysis is subject to biases related to data completeness and accuracy, with potential gaps in patient records affecting the consistency of the findings. Additionally, being a single-center study, the results may not be generalizable to other populations or healthcare settings where clinical practices and patient demographics might differ. The lack of a control group or comparison with alternative surgical techniques further limits the ability to generalize the results. Over the 20-year period, advancements in imaging technology and changes in surgical techniques could have introduced variability in diagnostic sensitivity and treatment outcomes, making it challenging to compare data across different time points. Furthermore, the lack of consistent long-term follow-up for all patients limits the ability to draw definitive conclusions about recurrence rates over 5 years of surgical efficacy. Despite these limitations, the study provides valuable insights into the management of cholesteatoma and emphasizes the need for standardized protocols and continued monitoring.

## 5. Conclusions

Our study offers a comprehensive evaluation of cholesteatoma management in patients with chronic otitis media, highlighting the effectiveness of tympanoplasty and mastoidectomy. The surgical success rate was high (81.89%), and 70% of patients experienced significant improvements in hearing following surgery, demonstrating the procedure’s positive impact on hearing restoration. However, severe complications were observed in 11.03% of patients, particularly following mastoidectomy. Patients with preoperative labyrinthine fistulae were more prone to complications, reinforcing the importance of thorough preoperative imaging. Preoperative ossicular damage also contributed to an increased risk of postoperative hearing loss, emphasizing the need for detailed assessment before surgery. Histopathological analysis revealed key insights into the cellular changes in cholesteatoma, with nearly half of the cases showing significant mucosal alterations. Immunohistochemistry identified T lymphocytes as the predominant immune cells, suggesting a robust inflammatory response in these patients. Despite the overall success, 1.55% of patients experienced recurrence within two years, underscoring the need for long-term follow-up and ongoing monitoring, particularly with advanced imaging techniques such as diffusion-weighted MRI.

In conclusion, successful cholesteatoma management requires a combination of precise surgical techniques, detailed imaging, and careful postoperative care. Further research is needed to explore the role of microbiota and biofilm in disease recurrence and to investigate potential molecular mechanisms that could improve treatment strategies.

## Figures and Tables

**Table 1:** CT aspects.

CT Aspects	Number of Patients	Percentage
Fluid accumulation	168	100%
Osteolysis	152	90.47%
Osteocondensations	128	76.19%
Cholesteatoma	48	28.57%
Exocranial extensions	48	28.57%
Intracranial extensions	24	14.28%
Changes to the external auditory canal	68	40.47%
Ossicular chain damage	144	85.71%

**Table 2:** MRI aspects.

MRI Aspects	Number of Patients	Percentage
Edema	128	100%
Cholesteatoma	40	31.25%
Exocranial extensions	40	31.25%
Intracranial extensions	32	25%
Inner ear damage	20	15.62%

**Table 3:** Hearing status at presentation.

Variable	Number (%)
Patients with hearing loss	480 (82.76%)
Type of hearing loss	Conductive: 340 (70.83%) Sensorineural: 140 (29.17%)
Mean preoperative pure tone average	55 dB ± 10 dB
ABG	Mean ABG: 25 dB

**Table 4:** Summary of surgical outcomes, complications, and recurrence rates with associated odds ratios and statistical analysis.

Variable	Number (%)	Odds Ratio (OR)	95% Confidence Interval (CI)	*p*-Value
Severe complications (post-mastoidectomy)	64 (11.03%)	10.81	3.89–29.96	<0.01
Postoperative complications (preoperative labyrinthine fistulae)	20 (3.45%)	3.5	1.5–6.9	<0.02
Post-operative hearing improvement (audiometry)	406 (70%)	5.38	4.1–7.0	<0.01
Post-operative hearing loss (preoperative ossicular damage)	70 (~12.07%)	2.8	1.4–5.6	<0.003
Recurrence in 2 years	9 (1.55%)	3.35	0.20–55.24	>0.08

**Table 5:** Postoperative hearing status.

Variable	Number (%)
Patients with improved hearing	406 (70%)
Patients with unchanged hearing	100 (17.24%)
Patients with worsened hearing	74 (12.76%)
Mean postoperative PTA	35 dB ± 8 dB
Improvement in PTA	≥10 dB improvement in 406 patients
Postoperative ABG	Mean ABG: 15 dB ABG ≤ 10 dB: 300 (51.72%) ABG > 20 dB: 120 (20.69%)
Patients with Complete Closure of ABG	180 (31.03%)

**Table 6:** Histopathologic aspects.

Histopathologic Aspects	Number of Patients
Cholesteatom	276
Polip	180
Tympanic membrane mucosa chamber, epithelium with apocrine-like cells, and chronic inflammatory infiltrate	128

**Table 7:** Cell types present in the inflammatory stromal infiltrate.

Cell Types	Percentage
Lymphocytes T	42.55%
Lymphocytes B	31.45%
Macrophages	26.00%

## Data Availability

Data are contained within the article.
